# Elucidating NO_*x*_ Surface Chemistry at the Anatase (101) Surface in TiO_2_ Nanoparticles

**DOI:** 10.1021/acs.jpcc.2c07489

**Published:** 2022-12-28

**Authors:** Lorenzo Mino, Marco Cazzaniga, Francesco Moriggi, Michele Ceotto

**Affiliations:** † Department of Chemistry and NIS Centre, University of Torino, Via Giuria 7, I-10125 Torino, Italy; ‡ Dipartimento di Chimica, Università Degli Studi di Milano, Via Golgi 19, I-20133 Milano, Italy

## Abstract

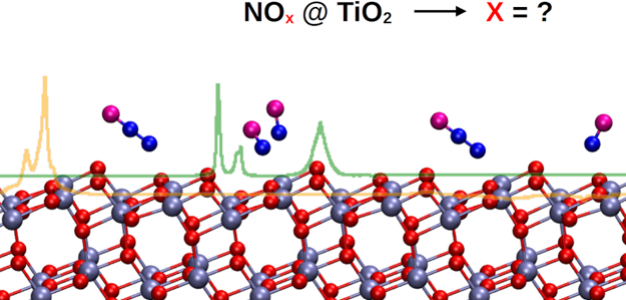

Understanding NO_*x*_ chemistry at titania nanoparticle surfaces is important for photocatalytic environmental remediation processes. We focus on this problem and put forward an experimental–computational approach based on vibrational spectroscopy grounds. Temperature-dependent IR experiments of NO_*x*_ adsorption on shape-engineered nanoparticle (101) anatase surfaces are paired with power spectra obtained from Born–Oppenheimer trajectories. Then, the harmonic versus anharmonic vibrational frequencies of several adsorption scenarios are directly compared with the IR experiments. We conclude that molecules are adsorbed mainly by the N-end side and both the intermolecular interactions between adsorbed molecules and (NO)_2_ dimer formation are responsible for the main NO adsorption spectroscopic features. We also investigate the spectroscopy and the mechanism of formation on defective anatase surfaces of the long-lived greenhouse gas N_2_O.

## 1 Introduction

Nitrogen oxides (NO_*x*_) are among the most troublesome atmospheric pollutant gases emitted during combustion.^1^ The most dangerous NO_*x*_ gases for human health and for the ecosystem are nitric oxide (NO), nitrogen dioxide (NO_2_), and nitrous oxide (N_2_O). In particular N_2_O is the third most important long-lived gas with strong greenhouse effects, given the ∼265 times atmospheric heat-trapping ability with respect to CO_2_, and one of the six greenhouse gases specified in the Kyoto Protocol.^2,3^ The major source of NO_*x*_ is road transport and about 80% of these emissions is due to diesel engines.^4^ The primary nitrogen-containing combustion product of vehicle engines is NO, which can be further oxidized to NO_2_.^5^ However, this conversion is not instantaneous and, depending on the meteorological conditions, can require few hours.^5^ Therefore, an efficient abatement of NO would considerably help also the reduction of NO_2_ pollution.^6^ To tackle this problem, photocatalytic NO degradation could be a promising approach.^7^ In this respect, TiO_2_ is the most studied semiconductor photocatalyst,^8^ and a better understanding of the surface processes involved in NO adsorption and subsequent transformations at its surface could help to design materials with improved photocatalytic performance.

The majority of the surface science studies about NO_*x*_ interactions with TiO_2_ present in literature deals with single crystals of rutile exposing the (110) surface,^9−12^ which is the most thermodynamically stable one.^13^ However, the anatase TiO_2_ phase, which is less stable in bulk form, is the most employed in photocatalytic applications involving nanoparticles (NPs); but for this polymorph, detailed surface science investigations are scarce. Many studies employed commercial anatase nanoparticles with an irregular morphology (e.g., the well-known TiO_2_ P25)^14−17^ and focused the attention on the reactivity of NO–O_2_ mixtures, which leads to the formation of NO_2_ and nitrites/nitrates.^18,19^ The evolution of these surface species under UV irradiation was also monitored employing spectroscopic techniques.^15,19,20^ Moreover, some researchers investigated the adsorption of N_2_O on the TiO_2_ surface,^21,22^ which is of interest to understand the reactivity of adsorbed NO, as we will clarify when discussing our results.

When studying these systems, many variables should be taken into consideration and it is unfortunately difficult to reach a definitive conclusion about the microscopic picture of the NO_*x*_ titania surface chemistry. Indeed, one should consider not only the possibility of different anatase facets but also of NP dimension, the surface defects, the presence of possible intermediate reactions in addition to the main photocatalytic process, and others. To this regard, the (110) rutile system may be more manageable but less photocatalytically interesting. In order to clear these issues, ab initio simulations have been performed in the past for NO_*x*_ adsorption on anatase.^22−27^ These simulations have been able to reproduce mainly minimum energy geometries, density of electronic states (DOS), or thermodynamics for different possible adsorption scenarios. This information has not been enough to clear the main issue of the NO_*x*_ adsorption, which is to provide a wide picture of the species involved in the adsorption process.

In this work, we employ both experiments and theory and compare them on the common ground of vibrational spectroscopy.^28^ This comparison will hopefully allow us to elucidate at the molecular level the surface chemistry of NO on the (101) anatase TiO_2_ facet, which is the most stable surface for the anatase polymorph and it is the dominant facet in the NPs usually employed in photocatalytic applications.^29^ To this end, we studied by Fourier transform infrared (FT-IR) spectroscopy the NO interactions with shape-engineered anatase TiO_2_ NPs, which show a slightly truncated bipyramidal shape, characterized by the overwhelming presence of {101} facets.^30^ This tailored surface engineering allowed us to minimize minority facets and defective randomness. We considered also NO co-adsorption and the N_2_O adsorption, which are crucial to rationalize the observed NO reactivity. The interpretation of the experimental results was assisted by density functional theory (DFT) simulations of the different NO_*x*_ adsorption configurations on the anatase (101) surface. In a first instance, the geometries and energetics of the NO_*x*_ adsorption has been calculated. Then, the vibrational frequencies for all the surface structures were calculated at the harmonic level and compared with the experimental IR signals, with a methodology already applied to other adsorbates.^31−35^ Eventually, we employed Born–Oppenheimer molecular dynamics (BOMD) to estimate the anharmonic contribution to each vibrational frequency. Our constant total energy (*NVE*) trajectories are at the zero-point energy shell value and for this reason, we call the approach “quasi-classical” approximation.^36^ We use the dynamical information originated from these trajectories to calculate the power spectrum, that is, the density of nuclear vibrational states, as obtained from the Fourier transform of the velocity autocorrelation functions.

## 2 Experimental Section

### 2.1 Materials and Characterization Techniques

#### 2.1.1 Preparation of Truncated Bipyramidal TiO_2_ Nanoparticles

The TiO_2_ nanoparticles (NPs), hereafter referred to as TiO_2_ bipy NPs, were prepared by forced hydrolysis of an aqueous solution of a Ti(TeoaH)_2_ complex (TeoaH = triethanolamine), using a concentration of 40 mM and an initial pH = 10, performing a hydrothermal treatment at 453 K for 90 h in autoclave. A more detailed discussion about the synthesis and characterization of these NPs, which show a truncated bipyramidal shape and a BET specific surface area of ∼40 m^2^ g^–1^, is reported elsewhere.^37,38^

#### 2.1.2 Electron Microscopy

Field-emission scanning electron microscopy (FESEM) images were acquired using a Tescan S9000G microscope, operating the instrument at 10 kV.

#### 2.1.3 FT-IR Spectroscopy

TiO_2_ NPs were pressed in self-supporting pellets (optical density of ca. 10 mg·cm^–2^) and placed in quartz cells, equipped with KBr windows, designed to carry out spectroscopic measurements at room temperature (RT) or at low temperatures (i.e., ∼100 K) by cooling with liquid N_2_. The cells were connected to a vacuum line (residual pressure < 10^–4^ mbar) allowing all thermal treatments and adsorption–desorption experiments to be performed in situ. Before NO and N_2_O adsorption, the samples were outgassed at 873 K for 120 min and contacted with 20 mbar of O_2_ at the same temperature. Then, the pellets were cooled to 373 K in O_2_ and then cooled to room temperature (RT) under outgassing. Infrared spectra were recorded on a Bruker Equinox 55 FT-IR spectrometer, equipped with an MCT cryogenic detector; 64 interferograms (recorded at 2 cm^–1^ resolution) were typically averaged for each spectrum.

### 2.2 Computational Methodology

All calculations were performed with the Quantum ESPRESSO suite,^39^ adopting the Perdew–Burke–Ernzerhof (PBE) functional and ultrasoft pseudopotentials with an energy cutoff of 60 Ry for the wavefunction and 480 Ry for the density. The anatase TiO_2_ (101) surface was modeled with 4 Ti atomic layers: the 2 deepest Ti layers were frozen to the bulk atomic position, while the others were relaxed to equilibrium geometry. The separation between periodic slab replicas has been achieved by inserting around 9 Å of vacuum. The Brillouin zone was sampled only at the Γ point. The resulting periodic system is composed of a supercell containing 48 atoms and exposing four fivefold-coordinated Ti adsorption sites (Ti_5c_). The adsorption geometry optimization procedure typically started with an intuitive initial geometry and it was followed by a full optimization process. This computational setup is a compromise between accuracy and feasibility. On the one side, the supercell is not too big for BOMD simulations which imply thousands of classical trajectory evolution time steps. On the other, we show below, as well as in a previous work for the adsorbed water molecules,^40^ how the harmonic frequency values do not change significantly for different supercell setups.

For NO and (NO)_2_ all calculations were spin polarized. Adsorption energies *E*_ads_ were calculated according to the following formula
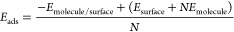
where *E*_surface_ is the energy of the bare TiO_2_ slab, *E*_molecule_ is the energy of the isolated molecule, and *N* is the number of adsorbed molecules. Harmonic vibrational frequencies were computed within the density-functional perturbation theory (DFPT) implementation available in Quantum ESPRESSO.^41^

We tested how much the geometry configurations and the harmonic frequency values are converged with respect to the supercell dimension in the case of the NO_*x*_ adsorptions. More specifically, we have checked the effect of additional reciprocal space points (see Supporting Information, Table S1) and increasing slab thickness (see Supporting Information, Table S2) to verify that these parameters do not significantly influence the calculated geometries and frequency values. Then, we also check below if the inclusion of dispersion and *U*-interaction terms are modifying the frequency values (see Supporting Information, Table S3). In the same table, the DFPT values are scrutinized by comparison with the finite difference (FD) approach for the calculation of the Hessian matrix. More specifically, the DFPT internal subroutine is based on a perturbative approach, while the finite difference approach directly calculates the ab initio Hessian matrix by finite differentiation of the atomic forces with respect to the nuclear displacement.

The non-analytic terms, which correct the long wavevector limit (*q* → 0) of the dynamical matrix for polar crystals, were neglected. A test on NO and N_2_O showed that this assumption has negligible effects on vibrational frequencies of the adsorbed molecule.

Quasi-classical anharmonic vibrational frequencies were estimated by the calculation of the time-averaged velocity–velocity autocorrelation function on a molecular dynamic trajectory initialized at the equilibrium geometries and initial velocities corresponding to the harmonic zero point energy (ZPE) motion.^42−44^ The Born–Oppenheimer molecular dynamics (BOMD) simulations were performed in the *NVE* ensemble; the atomic coordinates were evolved by means of a velocity Verlet algorithm for at least 10,000 MD steps of 10 a.u. each, yielding to 2.42 ps of dynamics. The time-averaged velocity–velocity autocorrelation function has been computed in normal mode coordinates for up to 10,000 MD steps. These calculations provide a classical power spectrum, which approximates the fundamental frequency transitions as the estimates of the characteristic vibrational frequencies. All modes are considered in the Fourier transform, including very low-frequency frustrated translational and rotational mode signals.

## 3 Results and Discussion

### 3.1 FT-IR Study of NO and N_2_O Adsorption on Shape-Engineered TiO_2_ Nanoparticles

**Figure 1:**
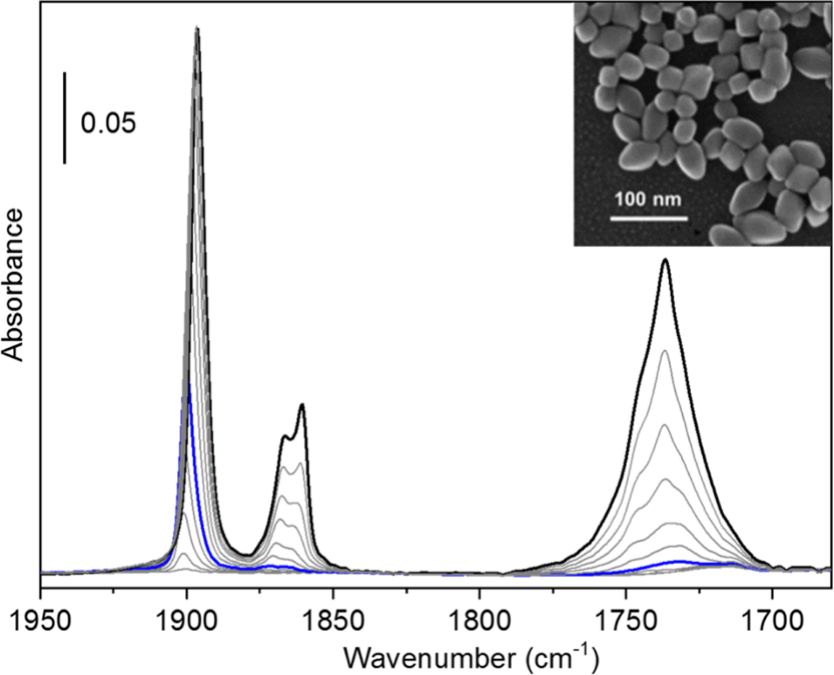
FT-IR spectra, acquired at ∼100 K, of NO adsorbed at increasing coverage on TiO_2_ bipy NPs, previously activated at 873 K. The spectrum of the activated material has been subtracted from all spectra. A FESEM image of the TiO_2_ bipy sample is shown in the inset.

A representative electron micrograph of the investigated anatase TiO_2_ bipy sample is shown in the inset of Figure 1. The NPs, which are obtained employing triethanolamine as a shape controller, appear as slightly truncated bipyramids with an average particle size of 45 ± 9 nm along the *c*-axis and 35 ± 5 nm in the *ab*-plane. The {101} facets account for ∼90% of the overall exposed surfaces. A comprehensive characterization of the morphological, structural, and surface properties of these samples is reported in previous publications.^30,38^

As the first step of our spectroscopic study, we analyzed the NO adsorption. NO, possessing an unpaired electron, is a free radical, very reactive, and prone to dimerization. Before starting the adsorption experiments, the TiO_2_ bipy NPs were outgassed at 873 K to remove the molecules adsorbed at the sample surface, which could influence the NO_*x*_ interaction.^45^ An oxidation treatment (see Section 2.1) was also performed to remove more effectively the organic contaminants. In the FT-IR spectrum collected after the activation (Figure S1 in the Supporting Information), the most intense signal at 2345 cm^–1^ is due to the antisymmetric stretching mode of slightly perturbed CO_2_ and weaker bands are also present in the 1600–1200 cm^–1^ spectral region, characteristic of carboxylates/carbonates groups. These features were already reported in literature in similar samples^46^ and are due to organic species, originated by the thermal decomposition of the titanium precursor, trapped in internal cavities (i.e. not influencing the adsorption experiments) present inside the NPs, as highlighted by previous HR-TEM studies.^30,38^ Conversely, the characteristic band of the water bending mode at 1620 cm^–1^ is not present and only very weak signals are visible around 3700 cm^–1^. These observations allow us to conclude that the main TiO_2_ facets are completely free from adsorbed molecular or dissociated water and only very few hydroxyls still remain on very undercoodinated sites, likely located in the NPs apical vertices.^47^ The FT-IR experiment was performed at ∼100 K because at room temperature only very low NO surface coverage can be investigated.^15,48^ Moreover, working at cryogenic temperatures allowed us to better discriminate between the adsorption and reactivity steps, as it will be discussed in details in the following. As visible in Figure 1, at low NO doses (up to the blue spectrum in Figure 1) a single IR peak is present, initially centered at 1901 cm^–1^ and gradually shifting till 1896 cm^–1^, showing a hypsochromic shift of 20–25 cm^–1^ with respect to the vibrational frequency of NO in the gas phase (1876 cm^–1^).^49^ At higher surface coverage, two new main signals start to grow in parallel: the former as a doublet at 1870/1860 cm^–1^, the latter as a broad absorption centered at about 1735 cm^–1^. In previous literature studies, these spectral features have been associated with different surface species, including bent NO, nitrosyls bonded to small amounts of Ti^3+^ cations, (NO)_2_, N_2_O_3_, and adsorbed HNO_2_.^14,18,19,50^ The comparison of our experimental data with the DFT results discussed in the next section is expected to contribute to clarify this issue. It is also worthy to note that working at low temperatures in very well controlled conditions we do not observe the appearance, as reported in previous studies, of other bands in the 2300–2200 cm^–1^ and/or 1600–1200 cm^–1^ spectral regions, which can be ascribed to the formation of N_2_O and/or nitrates.^15,18,51^

**Figure 2:**
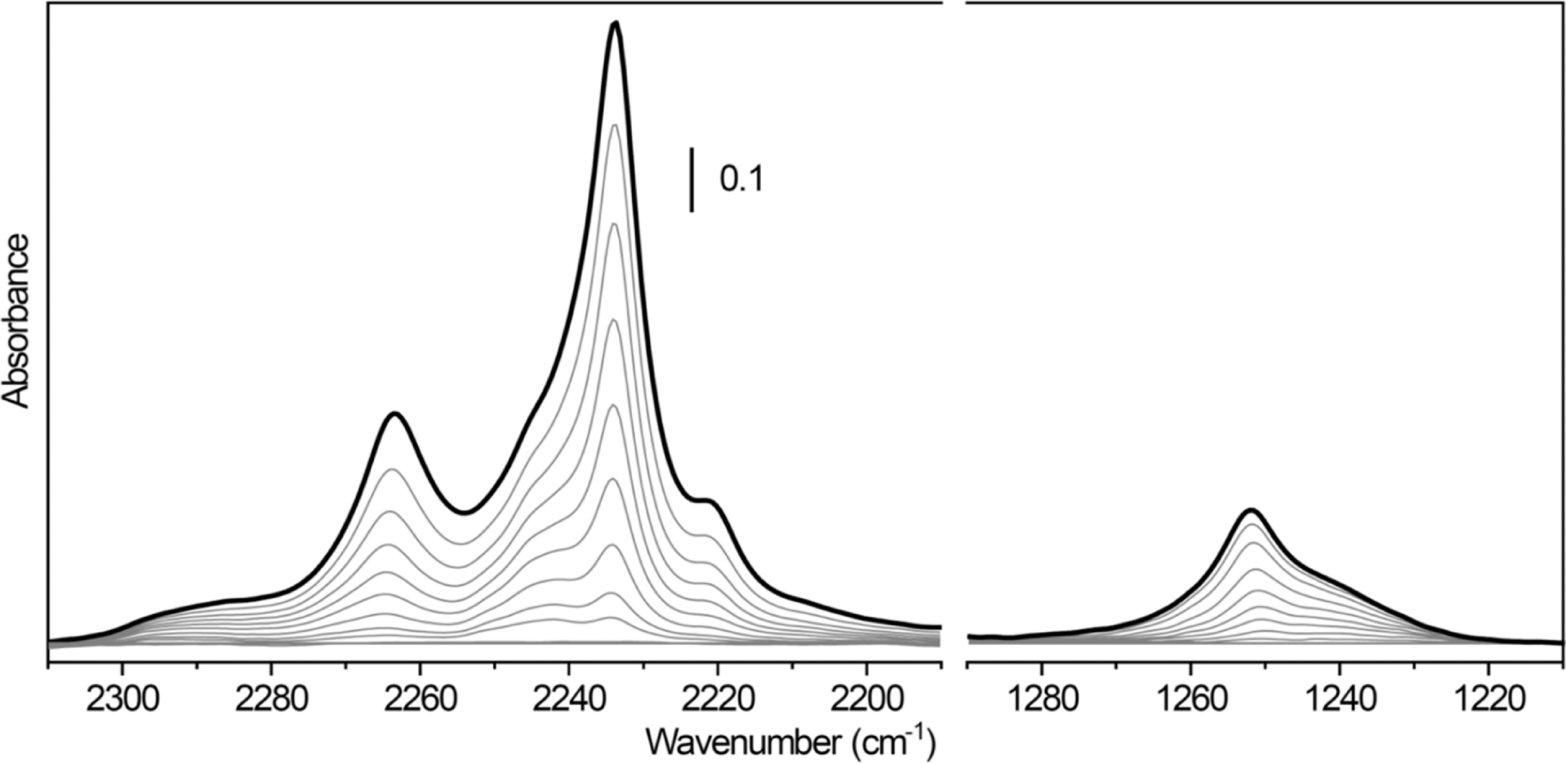
Room-temperature FT-IR spectra of N_2_O adsorbed at increasing coverage on TiO_2_ bipy NPs, previously activated at 873 K. The spectrum of the activated material has been subtracted from all spectra.

We moved, then, to the study of the N_2_O adsorption, which can be conveniently followed at room temperature. From Figure 2, we can see that, after admission of increasing N_2_O doses on the sample, two main spectral features grow in the high wavenumber region, centered at 2263 and 2234 cm^–1^, which can be assigned to the asymmetric stretching of N_2_O adsorbed with different surface configurations, as it will be shown by the following DFT simulations. In parallel, also a broad absorption, likely containing more than one component, appears at 1250 cm^–1^ and it may be associated with the symmetric stretching mode. In the gas phase, the N_2_O molecule shows also two degenerate bending modes occurring at 589 cm^–1^,^52^ which cannot be observed when N_2_O is adsorbed on the anatase surface because the corresponding spectral region is obscured by the TiO_2_ framework modes.

### 3.2 Modeling of NO_*x*_ Adsorption on the Anatase (101) Surface

In the following, we present a computational approach of the NO_*x*_ adsorption chemistry, aimed at explaining these FT-IR experimental results and providing additional physical insights.

#### 3.2.1 NO Adsorption

**Figure 3:**
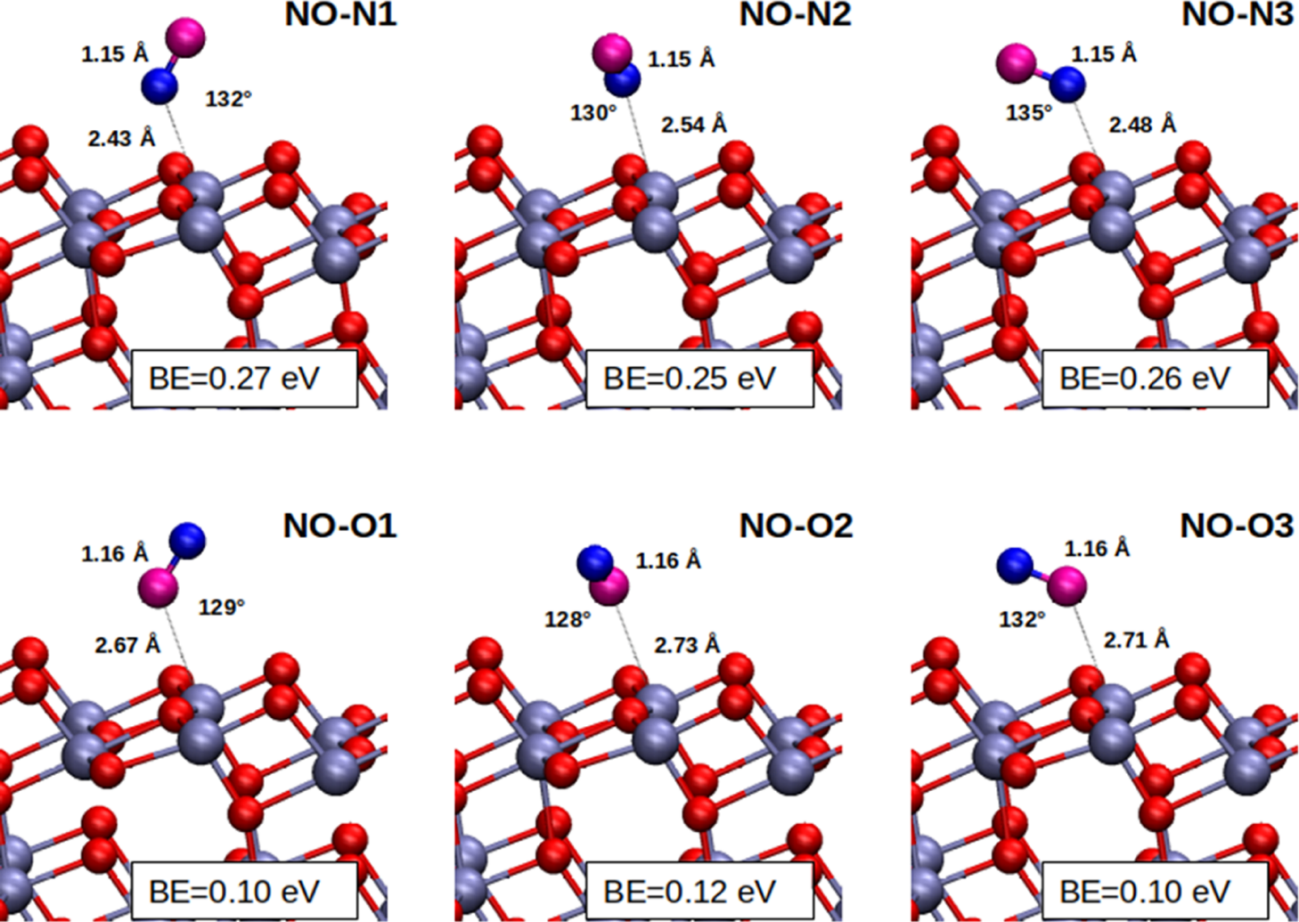
Different possible surface configurations for NO adsorption at θ = 0.25. N atoms are dark blue, O atoms of the NO molecule in violet and of TiO_2_ in red, while Ti atoms are light steel blue. N-end adsorption geometries are reported on the top row while O-end ones on the bottom. The binding energies in eV units for each adsorption configuration are reported.

We start our DFT study by analyzing NO adsorbed on anatase (101) at low coverage (θ = 0.25) and considering different possible adsorption geometries, as shown in Figure 3. The main geometrical parameters and the corresponding adsorption energies are reported in Table 1.

**Table 1:** Relevant Geometrical Parameters and Binding Energies for Gas Phase NO and for NO Adsorbed in Different Configurations on the TiO_2_ Anatase (101) Surface

structure	coverage	*d*(Ti_5c_–N) [Å]	*d*(N–O) [Å]	α(Ti_5c_–N–O) [°]	BE [eV]
gas phase			1.161		
Adsorption from N					
NO–N1	0.25	2.435	1.153	132	0.27
NO–N2	0.25	2.539	1.152	130	0.25
NO–N3	0.25	2.477	1.153	135	0.26
NO–N4	0.5	2.432	1.153	132	0.27
		2.432	1.153	132	
NO–N5	0.5	2.453	1.155	132	0.24
		2.453	1.155	132	
Adsorption from O					
NO–O1	0.25	2.666	1.160	129	0.10
NO–O2	0.25	2.735	1.160	128	0.12
NO–O3	0.25	2.709	1.162	132	0.10

The most stable NO surface configuration is the adsorption of the molecule via the N end over a fivefold-coordinated Ti surface site (Ti_5c_). Different local minima with comparable binding energies, ranging from 0.25 to 0.27 eV, are found and reported on the top row of Figure 3. These geometries are in agreement with the literature results.^23,53^ In all these surface structures, the angle *α*(Ti_5c_–N–O) is about 130°. This tilted adsorption behavior, which has been already highlighted in some previous studies,^36,53^ can be attributed to the presence of an unpaired electron in the NO molecule localized on the nitrogen atom, which leads to the formation of non-linear bonds. The most stable NO–N1 adsorption geometry has been checked for different DFT computational setups in Table S3, that is, by adding dispersion effects (DFT-D3) and the *U*-term correction (DFT + U), with *U* = 3.3 eV, as usually performed in titania calculations.^54^ The geometry results of NO–N1 of Table 1 are confirmed.

We tested also the adsorption from the oxygen end which appears to be less favored (see lower panels in Figure 3 and Table 1), with a binding energy more than halved with respect to the N end case, in line with literature data.^23,53^ Also in this case, we found three different local minima (NO–O1, NO–O2, and NO–O3 in Table 1 and Figure 3), which are energetically quasi-degenerate.

Afterward, we considered the effect of different NO coverages at the most stable surface geometry for θ = 0.25 (NO–N1) and θ = 0.50. These geometries and binding energies are reported in Table 1 and in the Supporting Information (Figure S2). We can observe a small decrease in binding energy at the highest coverage, otherwise we can say that geometries and energetic are quite invariant.

#### 3.2.2 (NO)_2_ Adsorption

The NO_*x*_ titania surface chemistry is not limited to the NO adsorption and for a realistic description of the experimental IR signal, we model also the adsorption of the NO dimer. Indeed, when two NO molecules are close enough on the surface, their 2π* orbitals, containing the unpaired electrons, tend to overlap and to form a weak σ bond.

**Figure 4:**
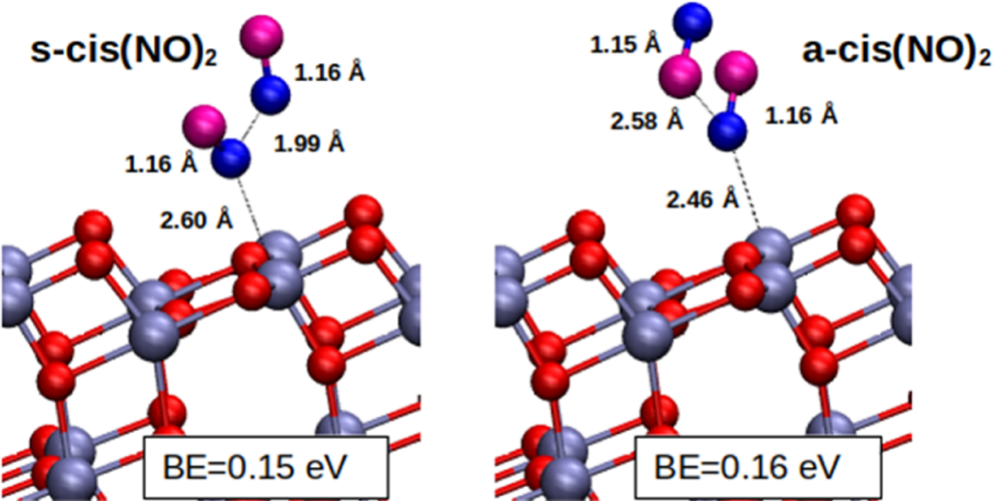
Optimized geometries for the NO dimers adsorbed in symmetrical, s-cis(NO)_2_ (left panel) and asymmetrical, a-cis(NO)_2_ (right panel) configurations. The binding energies in eV units for each adsorption configuration are reported.

A wide number of theoretical and experimental studies already analyzed (NO)_2_ and its ions in different phases and trapped in cryogenic matrices.^49,55,56^ Computational investigations pointed out the multiconfigurational character of (NO)_2_, which shows several minima with similar energy.^57,58^ DFT–PBE is somehow limited to describe accurately the electronic structure of these types of systems. Thus, we set back and compare our computational setup with other more accurate ab initio level of theories. Unfortunately, this type of comparison can be performed only for the gas phase, where some limitations are shown by the DFT approach as described more in details below. Consequently, our condensed phase DFT–PBE calculations provide mainly a qualitative picture. We considered two possible structures, which were proposed as the best candidates in a previous DFT study focused on the TiO_2_ rutile (110) surface.^9^ These are the cis(NO)_2_ species, where either a symmetrical or an asymmetrical configuration relative to the Ti_5c_ sites are possible. We denominate them, respectively, as s-cis(NO)_2_ and a-cis(NO)_2_^59^ and they are reported in Figure 4.

Table 2 shows how our DFT–PBE results agrees with other computational estimates in the literature at different levels of theory. While the agreement is strict with other DFT approaches, there is a difference with respect to the post-HF methods in terms of the nitrogen intramolecular distance estimates. In the following, we will check if this difference is significant also in terms of frequency, which is our main term of comparison with the experimental results.

**Table 2:** Relevant Geometrical Parameters and Binding Energies for (NO)_2_ in the Gas Phase and Adsorbed in Different Configurations on the TiO_2_ Anatase (101) Surface at θ = 0.25 Coverage

structure	method	*d*(N–N) [Å]	*d*(Ti_5c_–(NO)_2_) [Å]	BE [eV]
Gas Phase				
s-cis(NO)_2_	this work	1.999		
ref (55) s-cis(NO)_2_	B3LYP	1.991		
ref (53) s-cis(NO)_2_	CASPT2	2.327		
ref (52) s-cis(NO)_2_	CCSD(T)	2.123		
Adsorbed				
s-cis(NO)_2_	this work	1.987	2.603	0.15
a-cis(NO)_2_	this work		2.464	0.16

The adsorption energy of the dimer referred to the gas phase (NO)_2_ system is about 0.15 eV. To compare it with the NO adsorption energy, we consider that the binding energy of 2 NO molecules adsorbed on neighbor Ti_5c_ sites is 0.48 eV, which is equivalent to 0.24 eV per NO molecule (see Table 1). This is reasonable considering that both s-cis(NO)_2_ and a-cis(NO)_2_ interact with the surface only via a single N-end.

#### 3.2.3 N_2_O Adsorption

**Figure 5:**
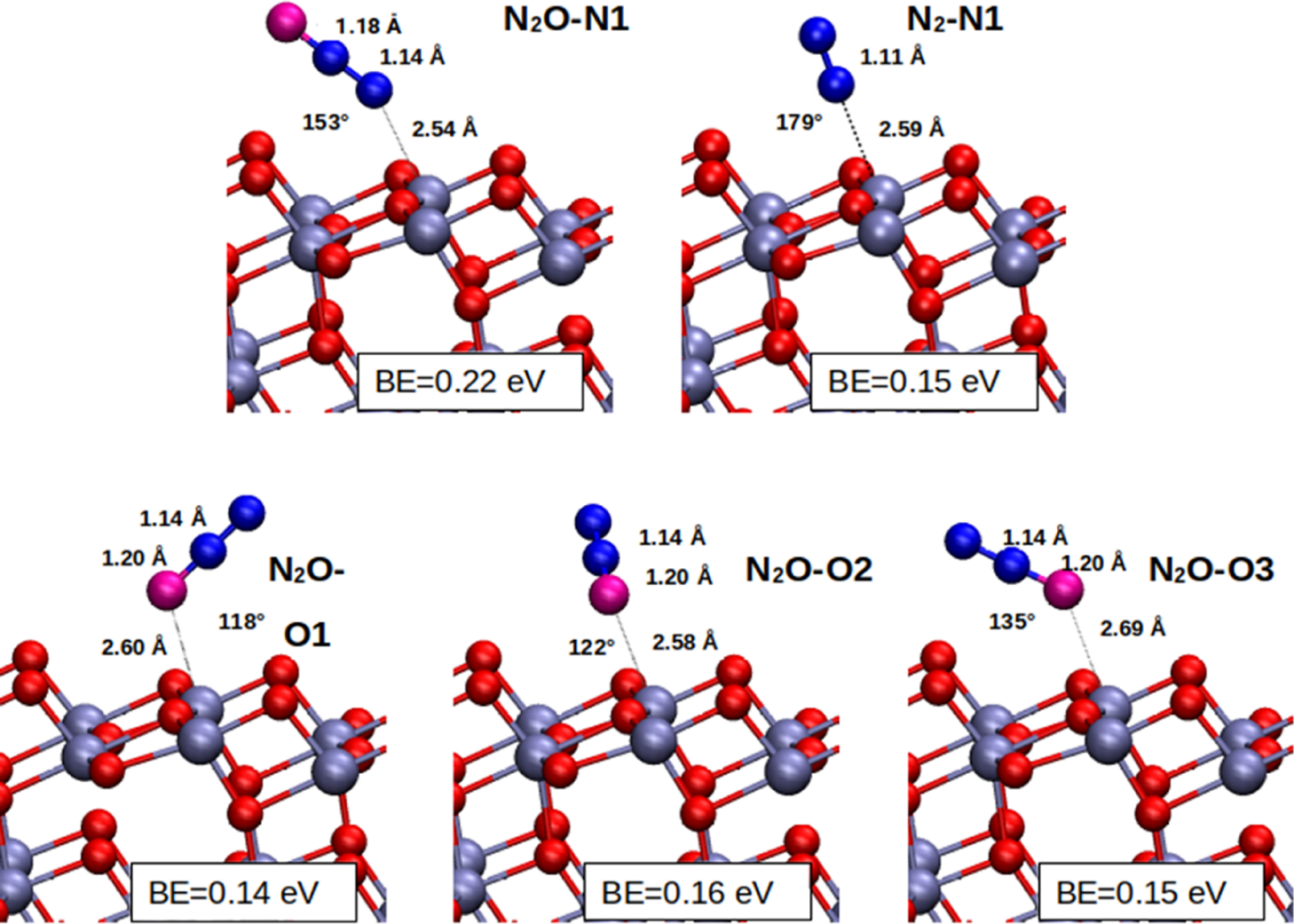
Different possible surface configurations for N_2_O adsorption at θ = 0.25 from the N-end (N_2_O–N1 structure) and from the O-end (N_2_O–O1, N_2_O–O2, and N_2_O–O3 structures). The binding energies in eV units for each adsorption configuration are reported. The N_2_ case is also reported for comparison.

Finally, we considered the N_2_O adsorption. The geometry optimizations have been performed in a spin unrestricted formalism as above. However, because this molecule is closed shell, the frequency calculations and BOMD simulations required by the quasi-classical approximation have been performed in a spin restricted formalism to save computational time, after checking that the geometries do not show significant differences compared with the unrestricted setup. As for the NO case, we tried different adsorption angles, finding only one stable configuration for adsorption from the N end (N_2_O–N1 structure in Figure 5 and Table 3). Also in this case, we tested for the lower energy geometry any variation with respect to dispersion corrections (DFT-D3) and *U*-term corrections (DFT + U). The data are reported in Table S3 and they confirm the results for the lower energy geometry shown in Table 3. We studied also the effect of coverage by simulating the N-side adsorption starting from the geometry at θ = 0.25 for the coverage θ = 0.50. A minimal decrease in adsorption energies is observed for different coverages, as reported by the N_2_O–N2 and N_2_O–N3 structures in Table 3. For each coverage setup, we reported the geometry information of each NO molecule in Table 3 and in Figure S3.

**Table 3:** Relevant Geometrical Parameters and Binding Energies for Gas Phase N_2_O and for N_2_O Adsorbed in Different Configurations on the TiO_2_ Anatase (101) Surface^a^

structure	coverage	*d*(Ti_5c_–N) [Å]	*d*(N–O) [Å]	*d*(N–N) [Å]	α(Ti_5c_–N–N) [°]	BE [eV]
gas phase N_2_O			1.190	1.141		
gas phase N_2_				1.108		
Adsorption from N						
N_2_O–N1	0.25	2.539	1.181	1.138	153	0.22
N_2_–N1	0.25	2.590		1.106	179	0.15
N_2_O–N2	0.5	2.554	1.181	1.138	152	0.22
		2.554	1.181	1.138	152	
N_2_O–N3	0.5	2.587	1.182	1.139	151	0.18
		2.587	1.182	1.139	151	

Adsorption from O						
structure	coverage	*d*(Ti_5c_–O) [Å]	*d*(N–O) [Å]	*d*(N–N) [Å]	α(Ti_5c_–O–N) [°]	BE [eV]
N_2_O–O1	0.25	2.599	1.197	1.136	118	0.14
N_2_O–O2	0.25	2.583	1.197	1.136	122	0.16
N_2_O–O3	0.25	2.689	1.196	1.136	135	0.15

a The N_2_ case is also reported for comparison.

Concerning the adsorption from the O side, we identified three different local minima (N_2_O–O1, N_2_O–O2, and N_2_O–O3 in Table 3 and Figure 5) with similar energy, in line with previous DFT results.^22^ Similarly to the NO case, for N_2_O the differences in binding energy between N-end and O-end adsorption is in favor of the N-end adsorption.

The geometry changes are also relevant. Comparing to the gas phase geometry, the adsorbed molecules show larger intramolecular bond distances. This effect suggests that these bonds are weaker after the adsorption process, as it is usually the case. For comparison, the nitrogen molecules are less perturbed in geometry after adsorption and the binding energy is smaller.

### 3.3 Anharmonic Vibrational Power Spectra and Comparison with Experiment

These calculations about the geometry and the energetic of the adsorbed NO_*x*_ did not allow us to reach a firm conclusion about the adsorption process. We now turn into the direct simulation of the vibrational spectra, in order to perform a direct comparison between the FT-IR experiments and the simulations.

**Figure 6:**
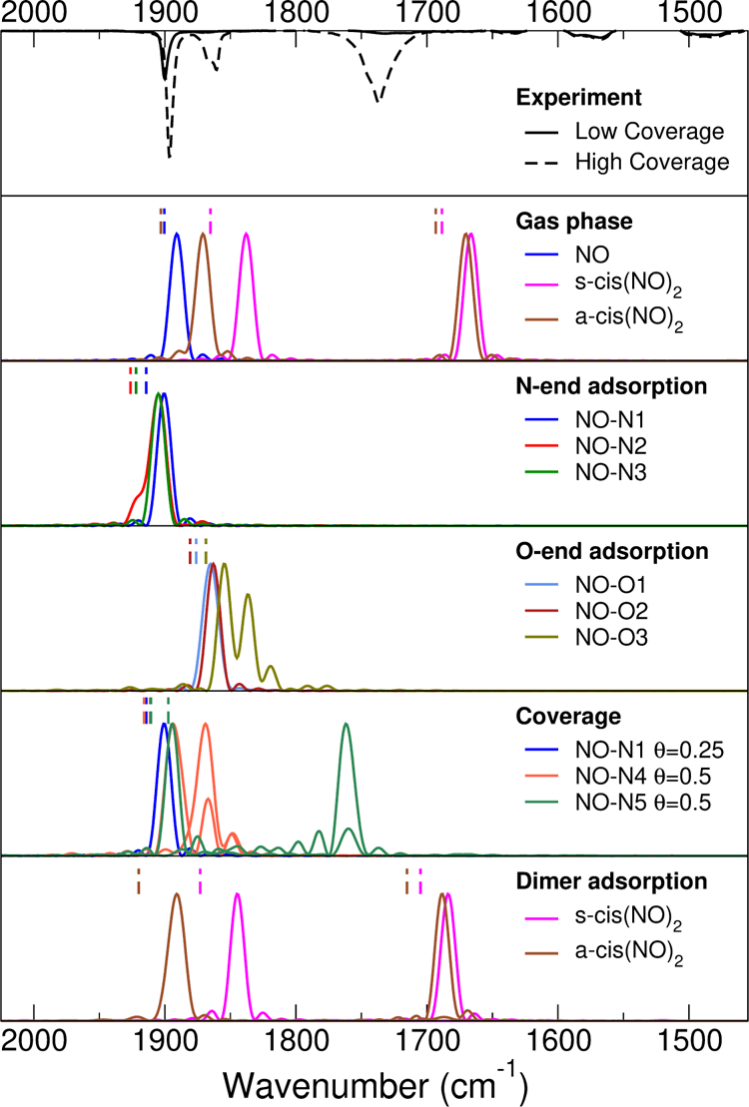
Quasi-classical power spectra for gas phase and different adsorption NO structures compared with experimental FT-IR spectra.

Figure 6 reports the simulated quasi-classical power spectra for the several different adsorption setups obtained in Tables 1 and 2, together with the experimental FT-IR spectra at high and low coverages reported on the top panel as a reference. On the second panel of Figure 6 one can find the gas phase NO anharmonic fundamentals obtained from our simulations of the isolated molecule and the dimer. The harmonic estimates are indicated as dashed vertical lines in the same panel. In the case of the gas phase NO molecule, the harmonic value is 1900 cm^–1^ and it is very similar to the anharmonic one (1891 cm^–1^), given the heavy atoms involved for this intramolecular stretch. The experimental value of the NO fundamental frequency stretch in gas phase is 1876 cm^–1^.^49^ This comparison shows that the PBE functional is slightly overestimating the experimental value. We keep this main limitation of the functional in mind, considering that higher levels of theory are out of reach for gas-surface BOMD simulations. However, in both simulated cases, the difference with the experiment is within 25 cm^–1^.

In the third panel of the same figure, the vibrational frequencies, both in the harmonic and quasi-classical approximations, of the NO adsorbed at different geometries are simulated. One can observe a small hypsochromic shift with respect to the gas phase, and a good agreement with the experimental results. The experimental IR band at 1896 cm^–1^ is reproduced by the quasi-classical frequency (1901 cm^–1^) and by the harmonic one (1914 cm^–1^), both referred to the lowest energy configuration (the NO–N1 structure in Figure 3). In the fourth panel, the O-end adsorption quasi-classical spectra are reported. These results may provide a partial explanation of the experimental side peaks at 1860 and 1870 cm^–1^. However, because the binding energy of these configurations is much smaller than the N-end ones, we think that this is not the explanation of the additional high coverage experimental peaks. Indeed, for all the O-end adsorption configurations, the NO vibrational frequency shows a bathochromic shift with respect to the molecule in the gas phase, which is not observed in IR spectra, further confirming that these structures are not present in our experiments. We think that a contribution to the experimental peaks at 1870 and 1860 cm^–1^ and of the wider one at 1730 cm^–1^ (with a shoulder at 1735 cm^–1^) should be attributed to the increasing coverage and to the concomitant dimerization of the adsorbed NO. For these reasons, we simulated the quasi-classical power spectra at higher NO coverage and separately for the s-cis(NO)_2_ and a-cis(NO)_2_ geometries presented in Figure 4. At increasing NO coverage, we can observe that the dynamics vibrational couplings, established when more adsorbed molecules are present and which is reproduced by the quasi-classical trajectories, causes the original stretching peak to split into many, depending on the coverage arrangements and amount. The more NO molecules are interacting, the higher is the number of splitting. However, in all cases, the splitting generates peaks, which are red-shifted with respect to the original stretching frequency, and this resembles the presence of the experimental features at 1870 and 1875 cm^–1^. We recall here that quasi-classical BOMD power spectra are of arbitrary intensities and a comparison with the experiments should not be performed on this ground but in terms of frequency only.

Because we think that the formation of the adsorbed NO dimer at increasing coverage is quite frequent, we simulated the spectra of the dimer for the geometries presented in Figure 4 and Table 2. The spectra are reported on the bottom panel of Figure 6. Indeed, the s-cis(NO)_2_ signals, which are located at 1845 and 1684 cm^–1^ are compatible with but not equal to the experimental bands at 1870 and 1730 cm^–1^. To clear this issue, we looked at the results for the gas phase dimers reported in the literature, which are at 1797 and 1960 cm^–1^ at the B3LYP level of theory,^60^ 1849 and 1789 cm^–1^ for CASPT2,^58^ and 1868 and 1733 cm^–1^ for CCSD(T).^57^ The gas phase experimental values are 1863 cm^–1^ for the ν_1_ symmetric stretch of the cis-ON–NO, 1776 cm^–1^ for the ν_5_ asymmetric stretch in cis-ON–NO, and 1689 cm^–1^ for the ν_5_ N=O asymmetric stretch in cis-ON–ON.^61^

When we compared all these data with our gas phase values (1865 and 1689 cm^–1^ at the harmonic level), we conclude that the DFT–PBE approximation describes reasonably the vibrational mode at a higher frequency (i.e., ν_1_). Conversely, the ν_5_ mode (*b*_2_ antisymmetric) is predicted to be too low in frequency by our computational setup, while it is better described by more sophisticated methods which are not, however, useful for gas-surface calculations. Upon dimer adsorption at the anatase (101) surface, the ν_1_ frequency slightly increases, in line with the experimental behavior, and the description of the ν_5_ mode is expected to be less accurate, as anticipated above. Actually, these values are not very much different from the gas phase ones. Finally, it is worth noting that the binding energy calculated for both s-cis(NO)_2_ and a-cis(NO)_2_ structures are similar and this is compatible with the coexistence on the anatase (101) surface of both adsorbed dimer configurations at high NO coverages. This hypothesis could also explain the broadness and complexity of the bands observed in our experimental spectra. Interestingly, infrared bands in similar spectral positions (i.e., 1876 and 1746 cm^–1^) have been recently observed by reflection–absorption infrared spectroscopy (RAIRS) upon low-temperature NO adsorption on rutile TiO_2_ single crystals exposing the (110) surface and assigned to bidentate s-cis(NO)_2_ species.^12^

All these data about the vibrational frequencies of the NO_*x*_ adsorption at different geometries and coverages are summarized in Table 4.

**Table 4:** Harmonic and Anharmonic Vibrational Frequencies for NO and (NO)_2_ Molecules in Gas Phase and Adsorbed at the Anatase (101) Surface

	harm	BOMD
NO Gas Phase		
NO	1900	1891
NO Adsorption		
NO–N1	1914	1901
NO–N2	1926	1905
NO–N3	1922	1905
NO–N4	1916	1894
	1911	1869
NO–N5	1911	1894
	1897	1762
NO–O1	1876	1865
NO–O2	1881	1863
NO–O3	1869	1855
Dimer Gas Phase		
s-cis(NO)_2_	1865	1838
	1689	1666
a-cis(NO)_2_	1903	1871
	1693	1670
Dimer Adsorption		
s-cis(NO)_2_	1873	1845
	1705	1684
a-cis(NO)_2_	1920	1891
	1715	1688

In this table, we can observe that most of the time the harmonic contribution is within 20 cm^–1^ higher than the anharmonic one. However, when vibrational coupling between adsorbates is present, the amount of anharmonicity can be more than a hundred of wavenumbers, making the anharmonic approach necessary. This is probably because the harmonic approximation is limited to a single point evaluation, while our anharmonic calculations are based on a molecular dynamics approach that can reproduce the shape of the potential out of equilibrium and the vibrational resonances between co-adsorbed molecules.^36,62^ In the case of the dimer adsorption, the discrepancy between harmonic and anharmonic frequency is always within 30 cm^–1^.

The harmonic frequency values for the lowest energy NO and N_2_O adsorption geometries has been tested for different DFT computational setups, that is, by adding dispersion (DFT-D3) and the *U*-term (DFT + U). These additional calculations that are reported in Table S3 confirm the values of Figure 6.

To summarize this part of the spectroscopic analysis of NO_*x*_ adsorption at different coverages, the experimental FT-IR spectra are possibly explained in all its band composition by including in addition to the bare NO adsorbed dimer, the signal originated by the increasing interactions between co-adsorbed NO molecules. Actually, we found that for long BOMD simulations of high coverage systems, the dimer is formed, and the two scenarios are de facto equivalent.

**Figure 7:**
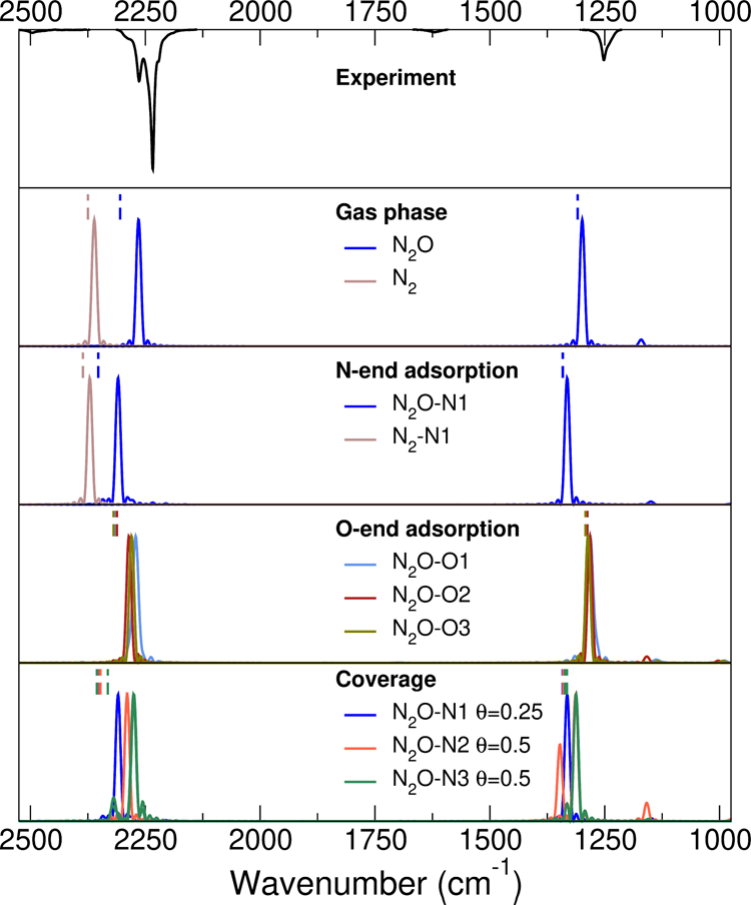
Quasi-classical power spectra for gas phase and different adsorption N_2_O structures compared with the experimental FT-IR spectra.

In the second part of our vibrational analysis, we look at the N_2_O molecular adsorption. Figure 7 reports on the top panel the FT-IR experiment for comparison. One can distinguish a spectroscopic feature around 2250 cm^–1^, composed of a weak shoulder at 2264 cm^–1^, an intense peak at 2234 cm^–1^ and another shoulder at 2220 cm^–1^, as already described in the experimental part above. Another band is located around 1250 cm^–1^. Gas phase simulations show two peaks, the ν_as_ = 2265 cm^–1^ and the ν_sy_ = 1299 cm^–1^ one. These values reproduce the experimental gas-phase N_2_O IR spectrum signals, respectively, at 2224 and 1285 cm^–1^.^63^ As before, the PBE functional is slightly overestimating the experimental values. The gas-phase simulated peaks are both hypsochromic shifted under N-end adsorption on the anatase (101) surface, respectively, at 2309 and 1332 cm^–1^. An analogous hypsochromic is observed for the O-end adsorption (N_2_O–O1 structure in Figure 5 and Table 3), but of a smaller entity. The concomitant presence of N-end and O-end N_2_O adsorption could explain the experiment features around 2250 cm^–1^ and that one at 1250 cm^–1^. However, we believe this is not the case, because the O-end adsorption binding energy is smaller than the N-end one, and the experimental peak that would be attributed to the O-end adsorption is much more intense. Instead, we believe that it is once again the increasing coverage and the dynamical couplings between adsorbates to be responsible for the experimental peak splitting around 2250 cm^–1^. To this end, we simulated on the bottom panel of Figure 7 different N_2_O adsorption setup spectra. Indeed, at increasing coverage the original ν_as_ stretching frequencies are red-shifted by an amount comparable with the experimental splitting. We think that the increased coverage explains also the broadness of ν_sy_ = 1250 cm^–1^ peak, as it can observe by comparison between the simulation and the experiment.

Harmonic and quasi-classical vibrational frequency values of Figure 7 are reported in Table 5. While the differences between harmonic and quasi-classical values for gas phase and low coverage are within 40 cm^–1^ between the two approaches, this discrepancy can be as large as 80 cm^–1^ when vibrational couplings between adsorbates kick in.

**Table 5:** Harmonic and Anharmonic Vibrational Frequencies for N_2_O and N_2_ Molecules in Gas Phase and Adsorbed at the Anatase (101) Surface

	harm	BOMD
N_2_O Gas Phase		
N_2_O	2305	2265
	1309	1299
N_2_	2375	2361
N_2_O Adsorption		
N_2_O–N1	2352	2309
	1341	1332
N_2_–N1	2386	2371
N_2_O–N2	2354	2290
	2348	2289
	1340	1313
	1339	1313
N_2_O–N3	2355	2276
	2332	2275
	1337	1312
	1332	1312
N_2_O–O1	2316	2271
	1287	1281
N_2_O–O2	2312	2286
	1288	1282
N_2_O–O3	2319	2280
	1292	1287

Finally, we investigated the isotope effect on the frequency values at harmonic level of accuracy (see Table S4) excluding any isotopic effects in the interpretation of the experimental spectra.

### 3.4 Vacancy-Mediated NO Conversion to N_2_O

**Figure 8:**
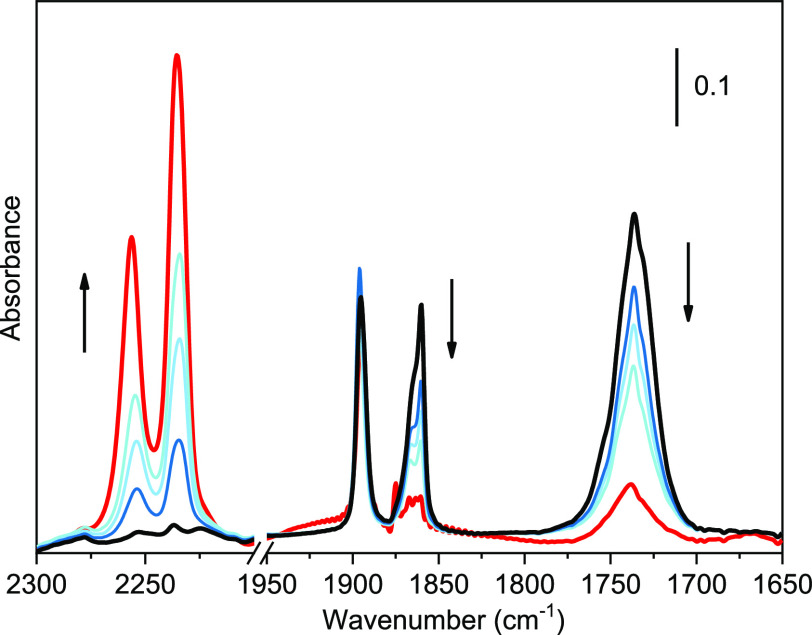
FT-IR spectra acquired while progressively increasing the temperature from ∼100 K (black spectrum) to ∼200 K (red spectrum) after adsorbing NO on TiO_2_ bipy NPs, previously activated at 873 K. The spectrum of the activated material has been subtracted from all spectra.

To complete our understanding of the chemistry of the NO_*x*_ titania adsorption, we investigate the temperature dependency of the experimental IR spectra to see if temperature triggers the formation of different NO_*x*_ species. We started our experiment with NO adsorbed at low temperatures on the TiO_2_ bipy sample (i.e., a surface condition similar to the black spectrum in Figure 1) and we progressively raised the temperature. As visible in Figure 8, we observe an expected progressive decrease in intensity of the bands centered at 1870/1860 and 1735 cm^–1^ associated with NO dimers, as explained above. At the same time, we notice the gradual growth of two components at 2260 and 2234 cm^–1^, which can be assigned to ν_as_ vibrations of N_2_O adsorbed in different surface configurations, as discussed in the previous section.

The experimental FT-IR spectra in Figure 8 is clearly suggesting the formation of the N_2_O species, as already observed in the literature,^50^ but the information about how the N_2_O is originated is still missing. We identified a possible mechanism for N_2_O formation mediated by a defective anatase surface, such as a surface oxygen vacancy. In fact, titania surfaces are most of time defective, that is, with the presence of oxygen vacancies in the first atomic layer and, more often, in the subsurface region.^64−66^ These defects are known to favor surface reactivity.^22,67,68^ Among the different possible processes, hereafter we addressed the vacancy of a surface O_2c_: although this it is not the most energetically favored and frequent defect, it establishes a reasonable path, even if it may not be the only one possible. Consequently, we run ab initio molecular dynamics simulations on the Born–Oppenheimer surface describing adsorbed NO molecules on the O-defective anatase (101) surface. The amount of kinetic energy in our on-the-fly *NVE* classical trajectories accounts for the experimental temperature raising, providing an estimation comparable to the higher temperature measurements. The simulations will allow us to see whether and how this kinetic energy is distributed and if, eventually, any bond is formed and/or broken.

**Figure 9:**
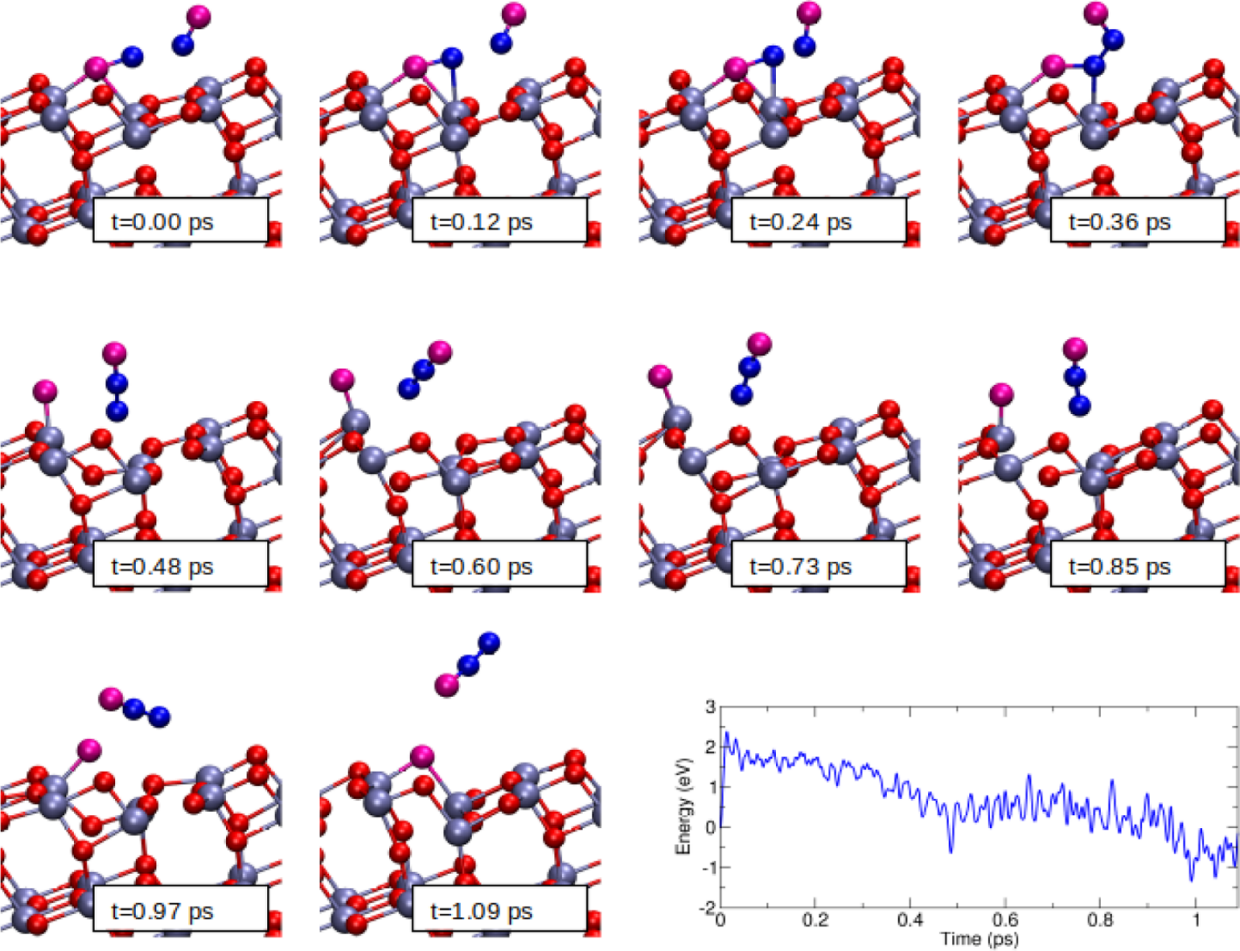
BOMD simulation snapshots and potential energy profile of the co-adsorption process of two NO molecules on O-defective anatase (101) surface. A N_2_O molecule is generated. For convenience, we set to 0 the energy corresponding to the MD starting configuration.

Figure 9 reports the snapshots of our ab initio trajectory by zooming on the regions where the NO molecules are adsorbed. Specifically, as reported on the *t* = 0 ps panel in Figure 9, one NO molecule has been adsorbed with the O-end on the O-defective surface site and another NO molecule is approaching the surface. The initial titania vibrational energy and the kinetic and vibrational energy of the NO molecules (which correspond to the ZPE of the system) are such that, converted in temperature, result in a value which is compatible with the higher temperature values of the FT-IR experiment of Figure 8. During the classical trajectory simulation, the kinetic and potential energy values describe the continuous exchange of energy between atoms. However, the total energy of the system is constant because of the classical trajectory evolution. In other words, we preferred to not employ any artificial thermostats but instead to simulate the natural evolution of the atomistic model by considering our classical trajectory setup as better representative of the co-adsorption physical process. In Figure 9, we can see how the initial NO projectile is orienting its N-atom toward the N-end of the adsorbed NO molecule. Subsequently, the N–N bond takes place, and an adsorbed (NO)_2_ complex is formed. At this point, the system could either be bounced back to its initial configuration, remain indefinitely adsorbed as (NO)_2_, or the NO bond of the original adsorbed molecule can be broken. The simulation indicates that this last path is taken by the trajectory and an N_2_O molecule is formed and desorbed. On the surface side, a stoichiometric titania surface is generated. In Figure 9 we reported also the energy profile of this trajectory, showing how the N_2_O formation is gradually reached. One may argue that a single trajectory is not representative of the process, which is composed of the many NO projectiles possible impinging energies and directions. To clear this issue, we performed a full dimensional system optimization starting from another possible configuration and reported in Figure S4. The optimization process reproduces the formation of N_2_O obtained at the end of the molecular dynamics simulation. However, additional simulations from different initial conditions show that the other possibilities, including the formation of the dimer, are contemplated and that the formation of the N_2_O molecule is just a possible route between other ones.

In conclusions, the concomitant temperature-dependent FT-IR spectroscopy and the DFT simulations show how the NO_*x*_ anatase surface chemistry includes also the production of the N_2_O molecule.

## 4 Conclusions

This paper describes the chemistry of NO_*x*_ compounds at the anatase (101) surface. To gain physical information, we set up an experimental and computational joint approach, which is based on the simulation of the vibrational frequencies. IR spectroscopy is a common ground between the theory and experiment that allows for direct comparisons to reach solid conclusions about the atomistic processes occurring at the oxide surface.

More specifically, on the experimental side, FT-IR spectra of NO_*x*_ adsorbed systems have been collected at variable temperatures on shape-controlled nanoparticles preferentially exposing anatase (101) surfaces. On the theory and computational sides, ab initio BOMD dynamics for classical power spectra calculations has been applied to the same system under several possible configurations.

Despite the limitations arising from the use of a DFT PBE functional, the comparison of the simulations with the experiments allowed us to conclude that the experimental spectroscopic features are mainly reproduced by including the interactions between adsorbates. Then, by comparing the harmonic versus the anharmonic quasi-classical ab initio BOMD frequencies, we conclude that the harmonic approximation is good for the frequency calculation of heavy atom intramolecular stretches, but significantly less accurate for intermolecular ones.

The advantage of a dynamics versus a single-point calculation is that the dynamical BOMD approach considers the possibility for several geometry configurations to be visited during the trajectory, given the low energy barriers present in these gas-surface type of systems, and properly include these in the frequency estimate. Figures S5 and S6 plot the adsorption dihedral angle value during the BOMD time steps for, respectively, the NO and N_2_O initial configurations (either N-end or O-end adsorbed) and the coverage values tested above. The figures show how the different minimum geometries in the harmonic calculations are actually entangled and the separation between different conformers is artificial. For example, a simulation at 0.5 coverage (not reported for the sake of brevity) can turn into a (NO)_2_ adsorption geometry one for a long enough BOMD simulation.

Other approaches could have been employed to calculate vibrational frequencies of molecular adsorbates,^69−74^ some of them including quantum effects and much more accurate than the present one, but they are mostly limited to lower dimensionalities and fitted potential energy surfaces (PESs). Most of the time, in surface chemistry, PES usage is not an option and direct dynamics ab initio approaches are the only possible viable tools.

We think that the present quasi-classical approach, which is based on on-the-fly ab initio simulations and where anharmonic effects are included but quantum mechanical ones not, can be a good choice when dealing with heavy atoms. Clearly, we would have preferred a quantum dynamics approach that we recently developed^36^ if lighter atoms, such as hydrogen ones, would have been involved.

In summary, we reach the conclusions that (i) the NO molecule is adsorbed on the N-end side; (ii) the NO molecule co-adsorbs and the strong interactions that are taking place between monomers and dimers are very important for the NO_*x*_ surface chemistry; and, finally, (iii) that, in the presence of O vacancies in the catalyst, the N_2_O molecule is generated and desorbed. Considering the notorious strong greenhouse properties of this molecule, we highlight the importance of finely tuning the titania stoichiometry to obtain efficient catalysts, which remove NO with minimal N_2_O production.

## Supporting Information Available

Dependence of the geometry and frequencies from the convergence parameters (*k*-point mesh and slab thickness); FT-IR spectrum of the TiO_2_ bipy NPs after outgassing at 873 K; figures of high coverage adsorption configurations of NO and N_2_O; dependence of geometry and frequencies from the DFT level; isotopic effect on harmonic frequencies; snapshots of a geometry optimization of two NO molecules in the presence of a vacancy; and values of the dihedra of NO and N_2_O adsorption along BOMD simulations (PDF)
