# Supplementary material for: Elucidating NOx Surface Chemistry at the Anatase (101) Surface in TiO2 Nanoparticles
Source: J Phys Chem C Nanomater Interfaces. 2022 Dec 28;127(1):437–49. doi: 10.1021/acs.jpcc.2c07489 (PMC9841571; doi:10.1021/acs.jpcc.2c07489)
Supplement: Supplementary file 1 — jp2c07489_si_001.pdf [file jp2c07489_si_001.pdf]

Supporting Information for:

Elucidating NO_x Surface Chemistry at the Anatase (101) Surface in TiO_2 Nanoparticles

Lorenzo Mino[a]*, Francesco Moriggi[b], Marco Cazzaniga[b], and Michele Ceotto[b]*

[a]Department of Chemistry and NIS Centre, University of Torino, Via Giuria 7, I-10125 Torino, Italy
[b]Department of Chemistry, Università degli Studi di Milano, Via Golgi 19, I-20133 Milan, Italy

*Corresponding authors e-mail addresses: lorenzo.mino@unito.it; michele.ceotto@unimi.it

Table S1. Relevant geometrical parameters, binding energies and harmonic frequencies computed with different k-point meshes for NO and N_2O adsorption on the (101) anatase surface.

K-points mesh	(Ti_{5c}-N) [Å]	d(N-O) [Å]	d(N-N) [Å]	α (Ti_{5c}-N-O) [°]	BE [eV]	Frequency [cm^{-1}]	
		**NO adsorption (NO-N1 geometry)**					
**1 x 1 x 1**	2.435	1.153		132	0.27	1914	
**2 x 2 x 1**	2.485	1.153		130	0.25	1920	
**4 x 4 x 1**	2.485	1.153		130	0.25	1921	
**6 x 6 x 1**	2.485	1.153		130	0.25	1919	
		**N_2O adsorption (N_2O-N1 geometry)**					
**1 x 1 x 1**	2.539	1.181	1.138	153	0.22	2352	1341
**2 x 2 x 1**	2.562	1.181	1.139	153	0.21	2350	1340
**4 x 4 x 1**	2.561	1.181	1.139	153	0.21	2350	1338
**6 x 6 x 1**	2.561	1.181	1.139	153	0.21	2350	1338

Table S2. Relevant geometrical parameters, binding energies and harmonic frequencies computed with different slab thickness for NO and N_2O adsorption on the (101) anatase surface.

Slab Thickness	(Ti_{5c}-N) [Å]	d(N-O) [Å]	d(N-N) [Å]	α (Ti_{5c}-N-O) [°]	BE [eV]	Frequency [cm^{-1}]	
			**NO adsorption (NO-N1 geometry)**				
**4 Layers**	2.435	1.153		132	0.27	1914	
**6 Layers**	2.448	1.153		131	0.26	1917	
**8 Layers**	2.450	1.153		131	0.25	1918	
			**N_2O adsorption (N_2O -N1 geometry)**				
**4 Layers**	2.539	1.181	1.138	153	0.22	2352	1341
**6 Layers**	2.565	1.181	1.138	154	0.20	2348	1335
**8 Layers**	2.568	1.182	1.138	154	0.20	2350	1335

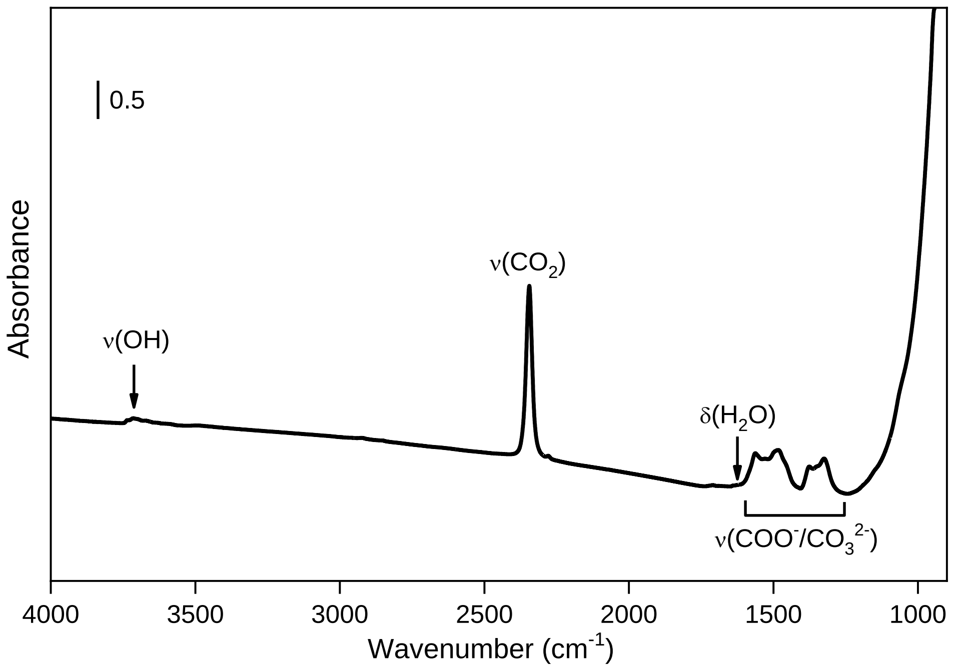

Figure S1. FT-IR spectrum of the TiO_2 bipy NPs after outgassing at 873 K. The main bands due to organic species originating from the decomposition of the Ti precursor and the expected positions of the signals of the water bending mode and OH stretching modes are highlighted.

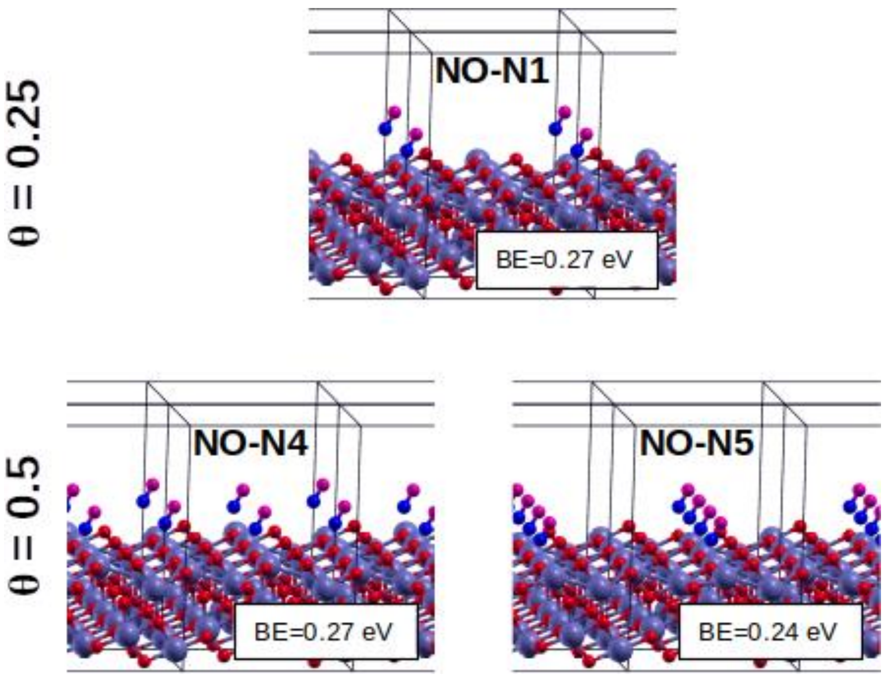

Figure S2. Different possible surface configurations for NO adsorption from the N-end as function of the coverage. Upper line $\theta = 0.25$ (NO-N1 structure); bottom line $\theta = 0.5$ (NO-N4 and NO-N5 structures).

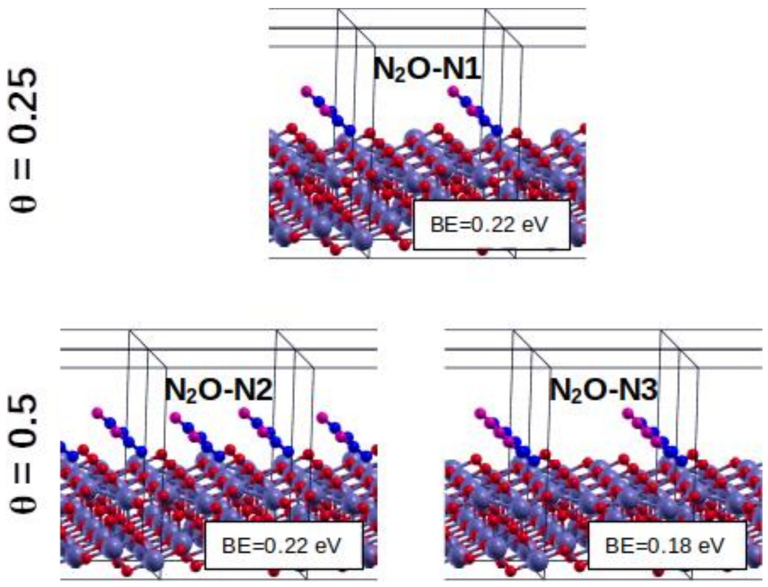

Figure S3. Different possible surface configurations for N_2O adsorption from the N-end as function of the coverage. Upper line $\theta = 0.25$ (N_2O-N1 structure); bottom line $\theta = 0.5$ (N_2O-N2 and N_2O-N3 structures).

Table S3. Relevant geometrical parameters, binding energies and harmonic frequencies computed with different level of theory for NO and N_2O adsorption on the (101) anatase surface. Atomic displacements of 0.005 Å have been used. DFPT: Density-Functional Perturbation Theory, FD: Finite Differences. The U term has been set to 3.3 eV.

Functional	Method	d(Ti_{5c}-N) [Å]	d(N-O) [Å]	d(N-N) [Å]	α (Ti_{5c}-N-O) [°]	BE [eV]	Frequency [cm^{-1}]	
			**NO adsorption (NO-N1 geometry)**					
**PBE**	DFPT	2.435	1.153		132	0.27	1914	
**PBE**	FD	2.435	1.153		132	0.27	1917	
**PBE+U**	FD	2.443	1.153		131	0.29	1916	
**PBE-D3**	FD	2.417	1.153		132		1917	
			**N_2O adsorption (N_2O -N1 geometry)**					
**PBE**	DFPT	2.539	1.181	1.138	153	0.22	2352	1341
**PBE**	FD	2.539	1.181	1.138	153	0.22	2352	1343
**PBE+U**	FD	2.533	1.180	1.139	152	0.24	2355	1346
**PBE-D3**	FD	2.523	1.180	1.138	149	0.44	2353	1344

Table S4. Isotopic effect on the harmonic frequencies for the most stable adsorption configurations of NO and N_2O.

	$^{14}N^{16}O$ Frequency [cm^{-1}]		$^{15}N^{16}O$ Frequency [cm^{-1}]		$^{14}N^{18}O$ Frequency [cm^{-1}]	
**NO adsorption (NO-N1 geometry)**	1914		1880		1864	
**N_2O adsorption (N_2O -N1 geometry)**	2352	1341	2278	1317	2344	1294

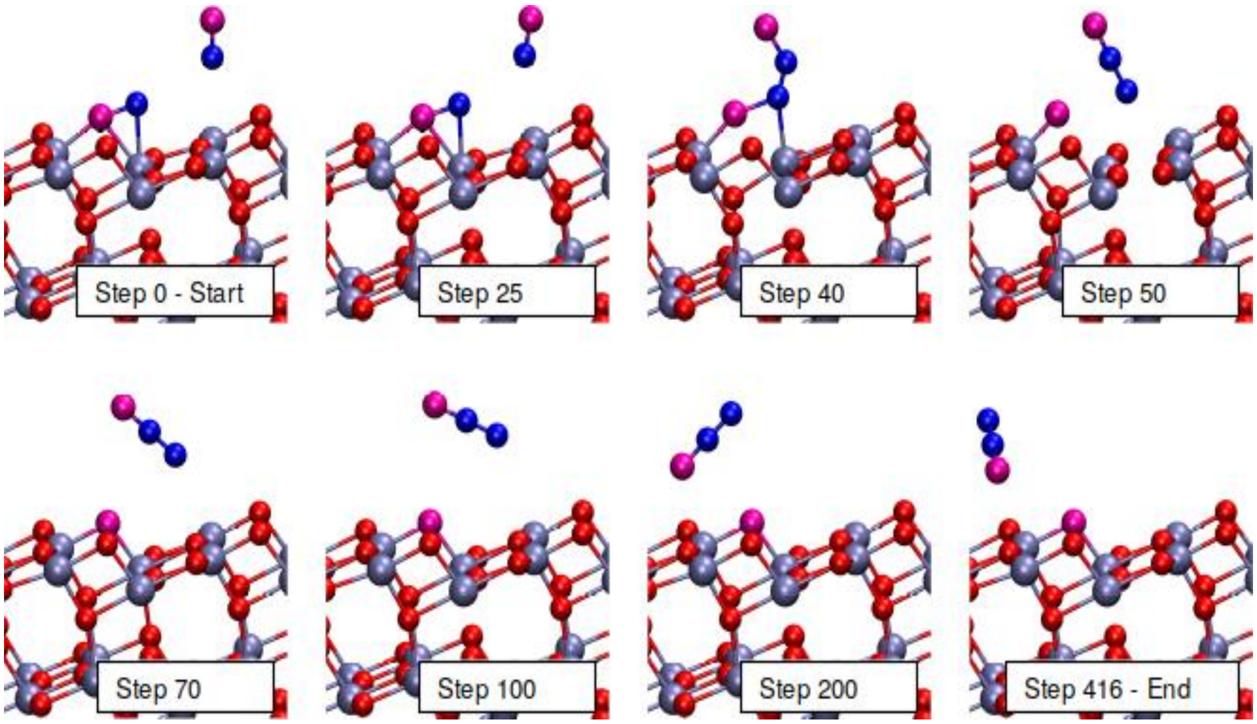

Figure S4. Snapshots of a geometry optimization for 2 NO molecules in presence of an oxygen vacancy.

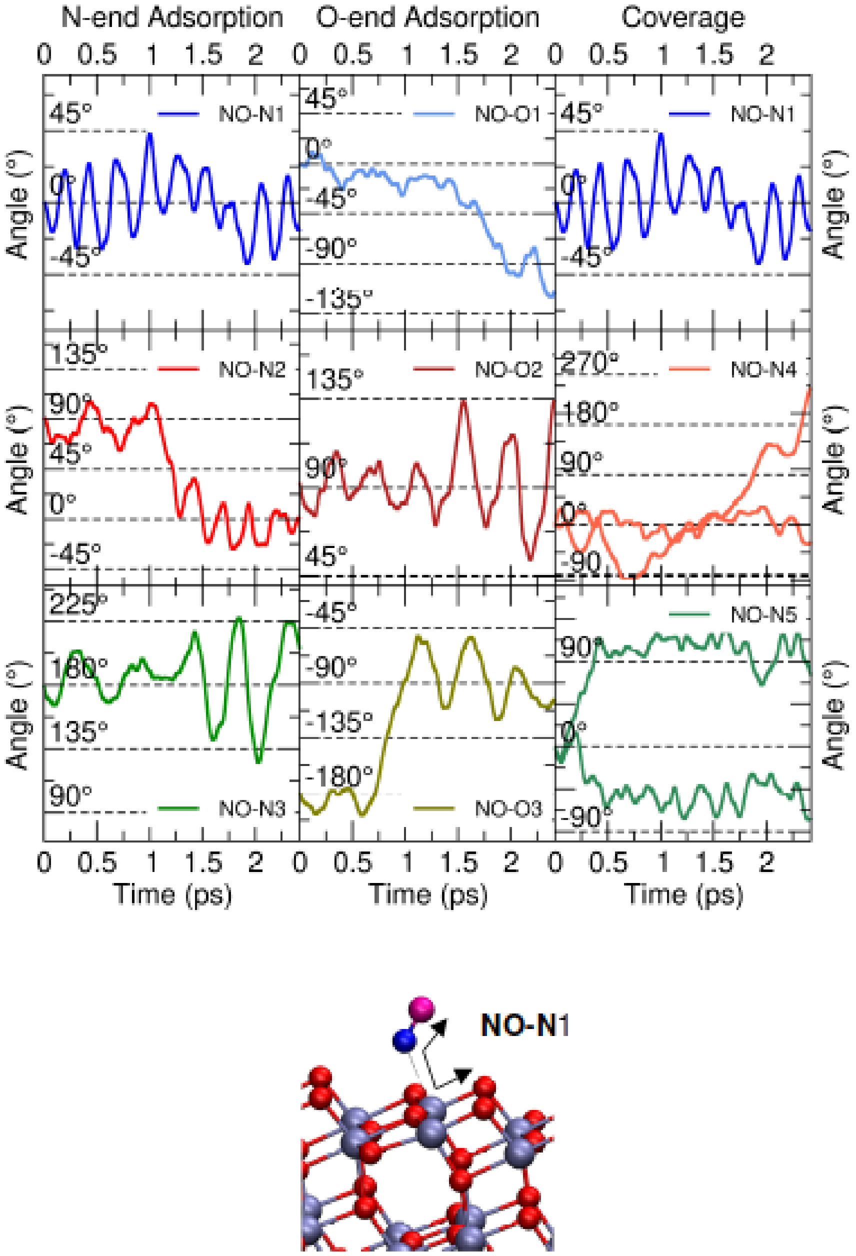

Figure S5. Dihedra of NO adsorption along BOMD simulation for different adsorption scenarios. First column for the N-end adsorption, second for the O-end and third column for the increasing coverage N-end adsorption.

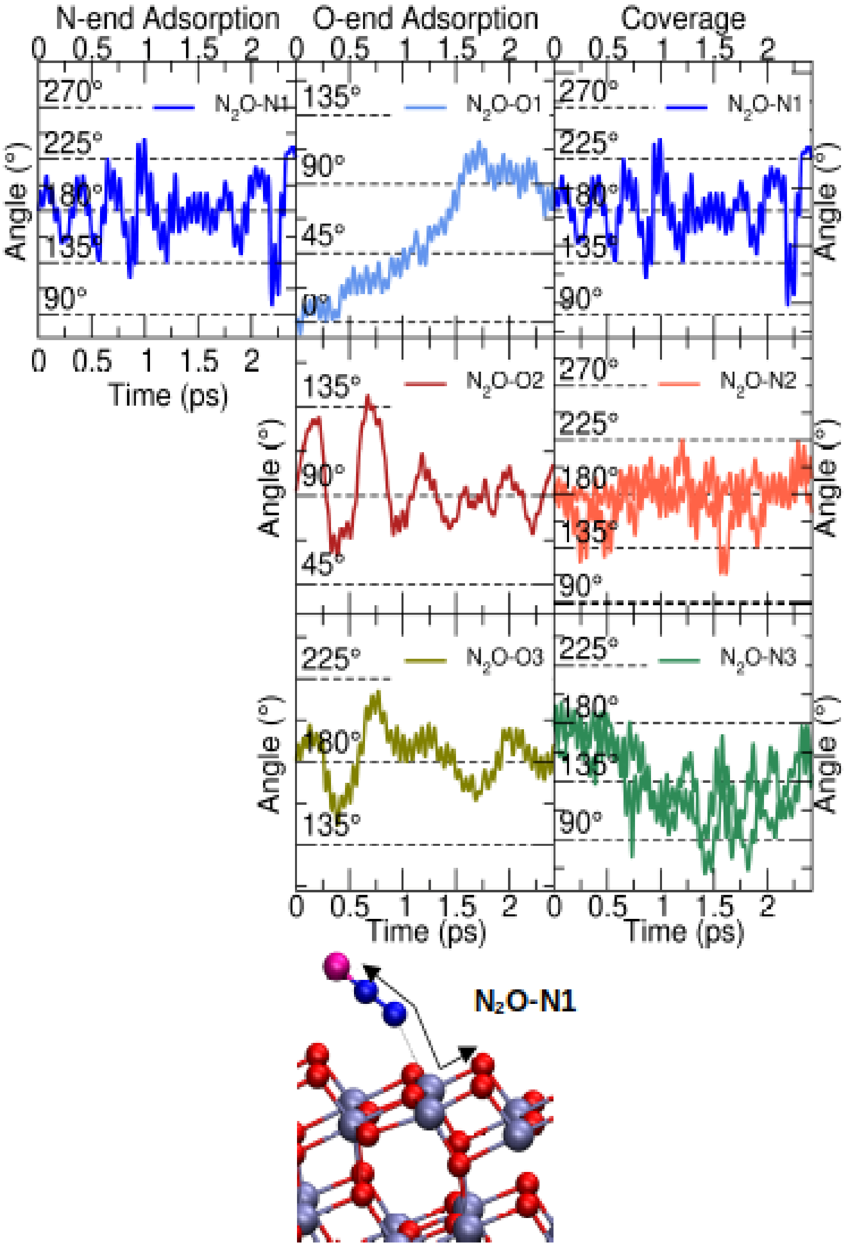

Figure S6. The same as in Figure S4 but for the dihedra of N_2O adsorption along BOMD simulation.
